# A Neonatal Unit Experience with Empiric Antibiotics for Late-onset Neonatal Sepsis: A Retrospective Study

**DOI:** 10.1097/pq9.0000000000000239

**Published:** 2019-12-09

**Authors:** Mountasser Mohammad Al-Mouqdad, Oluwaseun Egunsola, Sheraz Ali, Suzan Suahil Asfour

**Affiliations:** From the * King Saud Medical City, Ministry of Health, Riyadh, Saudi Arabia; † Clinical Pharmacology and Toxicology Research Group, Discipline of Pharmacology, School of Medical Sciences, Sydney Medical School, The University of Sydney, Sydney, NSW, Australia.

## Abstract

Neonatal sepsis remains a major cause of morbidity and mortality and warrants the immediate start of appropriate empiric treatment. Thus, this study compared the effectiveness of the 2 antibiotic regimens (cloxacillin–amikacin or cefotaxime–ampicillin) among neonates with late-onset neonatal sepsis.

**Methods:**

We conducted a retrospective cohort study comparing mortality between 2 treatment cohorts of very low birth weight neonates with late-onset sepsis, who had received amikacin–cloxacillin or cefotaxime–ampicillin between January 2014 and December 2017. There were 27 neonates in each treatment arm after 1:1 propensity score matching. Univariate analyses (Chi-square and independent *t* tests, where appropriate) were performed to determine the association between variables. We determined the hazard ratio for all-cause mortality using the Cox regression model.

**Results:**

We identified a total of 132 neonates from the hospital’s record. We included 27 neonates each in the amikacin–cloxacillin and cefotaxime–ampicillin groups. Intraventricular hemorrhage, necrotizing enterocolitis, birth weight, and gestational age were significantly associated with mortality (*P* < 0.05). The risk of mortality was significantly higher in neonates receiving empiric cefotaxime and ampicillin than those receiving amikacin and cloxacillin (hazard ratio: 2.91, 95% confidence interval: 1.17–7.30, *P* = 0.023).

**Conclusions:**

In our center, amikacin–cloxacillin combination therapy was associated with lower mortality in very low birth weight neonates with late-onset sepsis compared with cefotaxime–ampicillin therapy.

## INTRODUCTION

Sepsis is a leading cause of mortality and morbidity in neonates, particularly in developing countries,^1,2^ where about 30% of all neonatal deaths are attributed to severe bacterial infections.^3^ Approximately 750,000 deaths from neonatal sepsis occur annually worldwide.^4^ There is an inverse relationship between the incidence of neonatal sepsis and birth weight.^5,6^ The World Health Organization currently recommends ampicillin or cloxacillin (if the staphylococcal infection is suspected) and gentamicin for the empiric treatment of suspected neonatal clinical sepsis.^1^ However, third-generation cephalosporins, particularly cefotaxime, are also commonly utilized.

Late-onset neonatal sepsis is usually caused by neonatal intensive care units (NICUs)-acquired pathogens and changes in antimicrobial susceptibility patterns necessitate a regular review of antibiotics regimen. Gram-positive organisms, particularly coagulase-negative *Staphylococcus* and group B streptococci, are major causative agents of late- and early-onset neonatal sepsis, respectively.^7,8^ A recent study by Al-Mouqdad et al showed that the incidence of infection with Gram-positive microorganisms was 57% in a Saudi Arabian hospital’s NICU and coagulase-negative Staphylococcus was the predominantly isolated pathogen. They also reported that Gram-negative microorganisms, mainly *Escherichia coli*, *Klebsiella* sp., and *Pseudomonas aeruginosa*, accounted for 38% of isolated organisms.^9^ The study found that over 90% of the Gram-negative organisms were susceptible to gentamicin and amikacin.^9^ The United Kingdom’s Health Protection Agency’s national bacteremia surveillance also reported that over 95% of organisms causing neonatal sepsis were susceptible to gentamicin with either flucloxacillin or amoxicillin and amoxicillin with cefotaxime.^10^

Differentiating between neonatal sepsis and the symptoms of prematurity is challenging, as the symptoms of neonatal sepsis are nonspecific. Therefore, neonatologists prescribe broad-spectrum antibiotics under the assumption that neonatal sepsis exists, even after a negative result on initial blood culture.^11^ Evidence for the most suitable empirical antibiotics for late-onset neonatal sepsis is lacking;^12^ therefore, there are no consensus guidelines on an antibiotic regimen. Consequently, the empiric treatment of late-onset neonatal sepsis differs between NICUs and among countries. In our center, cloxacillin and amikacin are the commonly used antibiotics for the empiric treatment of late-onset sepsis. However, cefotaxime with ampicillin is also used occasionally.

On account of the increased survival of preterm neonates and their more extended hospitalization, late-onset sepsis will continue to be a challenge. In this study, we compared the difference in mortality between cloxacillin–amikacin and cefotaxime–ampicillin regimens in neonates with neonatal sepsis. We hypothesize that on account of better bacterial susceptibility to amikacin, empirical treatment of very low birth weight (VLBW) neonates with an amikacin-based regimen would result in lower mortality.

## METHODS

### Study Population and Setting

This study was carried out at a 70-bed NICU of a tertiary hospital in Saudi Arabia. We retrieved the electronic medical records of consecutively admitted VLBW neonates with first episodes of late-onset sepsis between January 2014 and December 2017. Only VLBW neonates (*<*1,500 g) with suspected late-onset neonatal infection were eligible for inclusion. We defined late-onset sepsis as sepsis occurring after 72 hours of birth. Demographic data including birth weight, gestational age, and sex were extracted, in addition to data regarding comorbid conditions such as intraventricular hemorrhage, necrotizing enterocolitis, hypoxic-ischemic encephalopathy, patent ductus arteriosus, and periventricular leukomalacia. We also extracted data regarding sedative and inotropic drug treatment during hospitalization; invasive respiratory support (ie, intubation) during hospitalization; the types of antibiotics received; the number of courses and the duration of antibiotics; and the number of days of hospitalization and the outcome of treatment. This study was approved (Reference number: H1RI-02-Jun16-01) by the institutional review board of the King Saud Medical City.

### Study Design

We performed a retrospective cohort study and identified 2 treatment cohorts. The primary cohort was neonates receiving empiric amikacin and cloxacillin for suspected late-onset sepsis. The comparison cohorts were neonates receiving empiric cefotaxime and ampicillin for suspected late-onset sepsis. A 1:1 propensity score matching of the 2 treatment groups to the nearest neighbor using the birth weights, gestational ages, need for respiratory support (received ventilatory support during hospitalization), and or central line support as the matching variables was carried out. We included respiratory and central line requirements in the matching variables because the choice of empiric antibiotics may be influenced by the clinical picture. The primary outcome of this study was the all-cause mortality during the first 120 days of life or discharge.

### Definitions

Clinical (suspected) sepsis: The exact definition of suspected neonatal sepsis remains vague. As the clinical features of sepsis may be influenced by strong pro-inflammatory cytokines, clinicians rely on clinical features in their decision to suspect sepsis and start antimicrobial agents.^13,14,15^

Confirmed (proven) sepsis: Detection of a pathogen (positive culture) in otherwise sterile body fluid, in addition to clinical and laboratory signs of sepsis.^13,14,15^

Early-onset sepsis: Sepsis caused by pathogens transmitted vertically from mother to infant occurring in the first 3 days of life.^13,14,15^

Late-onset sepsis: Sepsis caused by horizontally acquired pathogens that occurs after 3 days of an infant’s life.^14,16^

Blood sample for culture: We use standardized culture techniques to reduce false-negative results by taking 1-mL blood sample via venipuncture before starting antibiotics.

Narrow-spectrum antibiotics: These antibiotics include ampicillin, gentamicin, cloxacillin, and amikacin.

Broad-spectrum antibiotics: These antibiotics include any antibiotic not listed under narrow-spectrum antibiotics; we frequently use vancomycin, meropenem, and cefotaxime.

### Statistical Analysis

The baseline characteristics of both treatment groups were compared using Chi-square and independent *t* tests where appropriate. We used Cox regression to calculate adjusted hazard ratio. Statistical analyses were carried out using R version 3.4.3.

## RESULTS

We identified a total of 132 VLBW neonates who had received empiric amikacin–cloxacillin or cefotaxime–ampicillin for late-onset sepsis between January 2014 and December 2017. Thirty-six cases had reports of isolated microorganisms. The commonly isolated organisms include *Staphylococcus epidermidis* (21 cases), *Acinetobacter baumannii* (3 cases), and *Enterobacter cloacae* (3 cases) (Table 1), *E. coli* (2 cases), *Candida* sp. (1 case). One hundred and five neonates received amikacin–cloxacillin, whereas 27 received cefotaxime–ampicillin. The median duration of hospitalization among the patients was 45 (Interquartile range [IQR]: 31–71) days, and the duration of antibiotics during hospitalization was 16 (IQR: 9–26) days. The neonates received an average of 4 courses of antibiotics during hospitalization. After 1:1 matching of neonates in both treatment groups, we included 27 neonates each in the amikacin–cloxacillin and cefotaxime–ampicillin groups. Of those included in the study, 53.7% (29/54) were females. The mean gestational age of all the neonates in the study was 27.2 weeks, and the mean birth weight was 918 g. Table 2 describes the general characteristics of neonates in each of the treatment groups. Univariate analyses indicated a significant association of intraventricular hemorrhage, necrotizing enterocolitis, birth weight, and gestational age with mortality (*P* < 0.05). Hence, we used a Cox regression model that included all significant variables. The risk of mortality was significantly higher among neonates who received empiric cefotaxime and ampicillin compared with those who received amikacin and cloxacillin (hazard ratio: 2.91, 95% confidence interval: 1.17–7.30, *P* = 0.023) (Table 3).

**Table 1.**
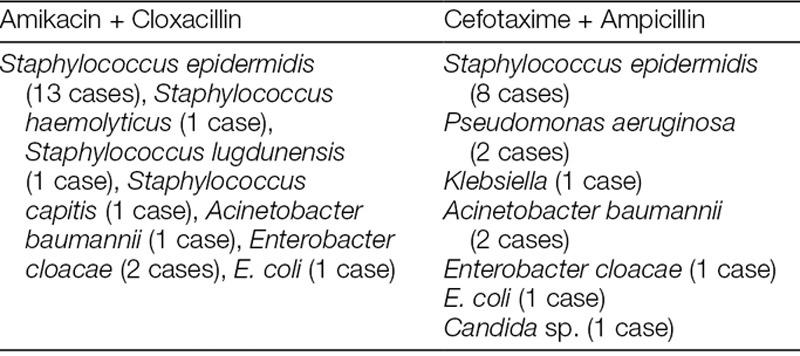
List of Cultured Organisms

**Table 2.**
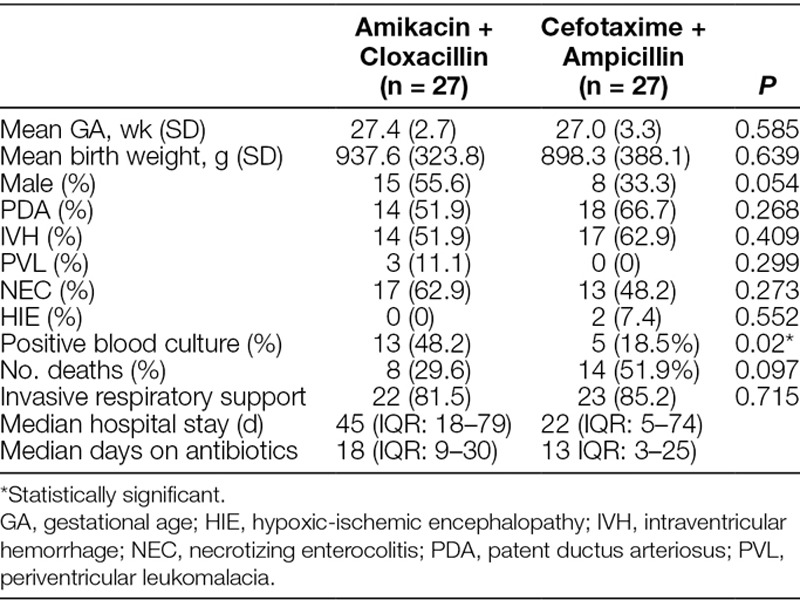
General Characteristics of Neonates in Both Treatment Groups

**Table 3.**
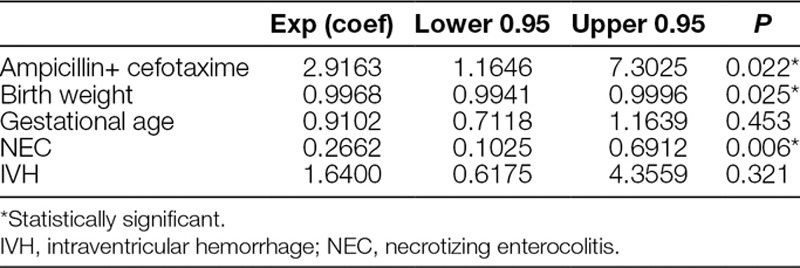
Multivariate Cox Regression Model

## DISCUSSION

In this study, we report a lower risk of mortality among neonates receiving empiric amikacin–cloxacillin for late-onset neonatal sepsis, compared with cefotaxime–ampicillin treatment. Cloxacillin, cefotaxime, and ampicillin are generally protective for Gram-positive bacteria and amikacin and cefotaxime for Gram-negative organisms. However, cefotaxime may not always provide sufficient Gram-negative antibacterial coverage, with only about 75% of the *Enterobacteriaceae* other than *E. coli* and 46% of the *Pseudomonas* spp. susceptible in the United Kingdom.^10^ Several studies have reported an increased risk of fungal infection, death, and neurodevelopmental delay with starting cefotaxime in the first few days of life.^17–22^ Clark et al reported a higher mortality rate among infants who had received cefotaxime in the first week of life, compared with gentamicin.^18^ Resistance to cefotaxime is increasing on account of extended-spectrum beta-lactamase infections, commonly by *A. baumannii*, *K. pneumonia*, and *E. coli.*^23^ Extended beta-lactamase infection account for approximately 15% of all neonatal infections in Saudi Arabia.^24–26^

Coagulase-negative *staphylococci* (CoNS) are the most frequent bacteria isolates in late-onset neonatal sepsis.^8^ In some countries, gentamicin–flucloxacillin or gentamicin–amoxicillin/penicillin is often adequate.^10,27^ However, in Saudi Arabia, the causative organisms and their antibiotic susceptibility patterns are different,^9,28,29^ thereby justifying the selection of other first-line empiric antibiotics. Although CoNs are often susceptible to vancomycin, targeted empiric therapy with vancomycin is usually reserved for neonates with the highest risk of severe infections.^11^ An ideal antibiotic combination regimen should cover the most frequently isolated organisms, without causing selection pressure for antibiotic resistance. The strategies adopted by various NICUs to prevent and treat late-onset neonatal sepsis may influence the pattern of bacteria, causing sepsis in the respective units. For example, the use of cefotaxime–amoxicillin in an NICU may increase the risk of cefotaxime resistance to Gram-negative organisms.^29^ Thus, with ampicillin–cefotaxime, Gram-negative bacteria like *Enterobacter* spp. may flourish following the elimination of normal intestinal flora. These organisms may degrade cefotaxime and cause cefotaxime-resistant invasive infections.^17^ In a previous study carried out at our center, enterobacter sepsis accounted for about 19% of neonatal sepsis.^9^ Therefore, it is prudent to consider these organisms when empiric therapy is prescribed for sick patients, those with previous positive blood culture, or following a prolonged course of antibiotics.

It is noteworthy that the selection of antibiotics in this study was not informed by a change in practice over time; rather, it was based on the hospital guidelines premised on the factors mentioned earlier. The first-line empiric treatment for late-onset sepsis in our center is a combination of amikacin and cloxacillin. Consequently, more neonates received this regimen compared with ampicillin–cefotaxime. This study, to our knowledge, is the first to compare empiric amikacin–cloxacillin with cefotaxime–ampicillin for late-onset neonatal sepsis. The propensity matching ensures an adequate comparison of both treatment groups. However, the study is limited by the small sample size, which limits the generalizability of the findings. Hence, the result should be interpreted with caution. Furthermore, we do not maintain a central line-associated bloodstream infection database at our center. Thus, we are unable to account for the effect of these infections on mortality.

In conclusion, amikacin–cloxacillin combination therapy was associated with lower mortality in neonates with late-onset sepsis at our NICU, compared with cefotaxime–ampicillin. Other important influences that can impact mortality need to be investigated in future studies. Therefore, a randomized controlled trial, which is likely to have better internal validity, is essential to determine the true effects of the treatment regimens. To control the misuse of antibiotics, institutions need to develop clearer guidelines based on antibiotics prevalence and susceptibility patterns.

## DISCLOSURE

The authors have no financial interest to declare in relation to the content of this article.

## References

[1] Fuchs A Bielicki J Mathur S Antibiotic sepsis use for in neonates and children: 2016 evidence update. WHO reviews. 2016:1–21.

[2] Liu wL Oza S Hogan D Global, regional, and national causes of under-5 mortality in 2000-15: an updated systematic analysis with implications for the sustainable development goals. Lancet. 2016;388:3027–3035. 27839855 10.1016/S0140-6736(16)31593-8 PMC5161777

[3] Seale AC Blencowe H Zaidi A; Neonatal Infections Estimation Team. Neonatal severe bacterial infection impairment estimates in South Asia, sub-Saharan Africa, and Latin America for 2010. Pediatr Res. 2013;74(Suppl 1):73–85. 24366464 10.1038/pr.2013.207 PMC3873707

[4] Ranjeva SL Warf BC Schiff SJ Economic burden of neonatal sepsis in sub-Saharan Africa. BMJ Glob Health. 2018;3:e000347. 10.1136/bmjgh-2017-000347 PMC5859806 29564153

[5] Stoll BJ Hansen N Fanaroff AA Late-onset sepsis in very low birth weight neonates: the experience of the NICHD Neonatal Research Network. Pediatrics. 2002;110(2 Pt 1):285–291. 12165580 10.1542/peds.110.2.285

[6] Stoll BJ Gordon T Korones SB Late-onset sepsis in very low birth weight neonates: a report from the National Institute of Child Health and Human Development Neonatal Research Network. J Pediatr. 1996;129:63–71. 8757564 10.1016/s0022-3476(96)70191-9

[7] Berardi A Rossi C Lugli L; GBS Prevention Working Group, Emilia-Romagna. Group B streptococcus late-onset disease: 2003-2010. Pediatrics. 2013;131:e361–e368. 23296441 10.1542/peds.2012-1231

[8] Jiang JH Chiu NC Huang FY Neonatal sepsis in the neonatal intensive care unit: characteristics of early versus late onset. J Microbiol Immunol Infect. 2004;37:301–306. 15497012

[9] Al-Mouqdad MM Alaklobi FA Aljobair FH A retrospective cohort study patient chart review of neonatal sepsis investigating responsible microorganisms and their antimicrobial susceptibility. J Clin Neonatol. 2018;7:141–145.

[10] Muller-Pebody B Johnson AP Heath PT; iCAP Group (Improving Antibiotic Prescribing in Primary Care). Empirical treatment of neonatal sepsis: are the current guidelines adequate? Arch Dis Child Fetal Neonatal Ed. 2011;96:F4–F8. 20584804 10.1136/adc.2009.178483

[11] Russell AB Sharland M Heath PT Improving antibiotic prescribing in neonatal units: time to act. Arch Dis Child Fetal Neonatal Ed. 2012;97:F141–F146. 21037285 10.1136/adc.2007.120709

[12] Gordon A Jeffery HE Antibiotic regimens for suspected late onset sepsis in newborn infants. Cochrane Database Syst Rev. 2005;20:CD004501. 10.1002/14651858.CD004501.pub2 PMC8665451 16034935

[13] Wynn JL Defining neonatal sepsis. Curr Opin Pediatr. 2016;28:135–140. 26766602 10.1097/MOP.0000000000000315 PMC4786443

[14] Metsvaht T Ilmoja ML Parm Ü Comparison of ampicillin plus gentamicin vs. penicillin plus gentamicin in empiric treatment of neonates at risk of early onset sepsis. Acta Paediatr. 2010;99:665–672. 20096030 10.1111/j.1651-2227.2010.01687.x

[15] Hornik CP Fort P Clark RH Early and late onset sepsis in very-low-birth-weight infants from a large group of neonatal intensive care units. Early Hum Dev. 2012;88(Suppl 2):S69–S74. 22633519 10.1016/S0378-3782(12)70019-1 PMC3513766

[16] Tzialla C Borghesi A Perotti GF Use and misuse of antibiotics in the neonatal intensive care unit. J Matern Fetal Neonatal Med. 2012;25(Suppl 4):35–37. 10.3109/14767058.2012.714987 22958010

[17] de Man P Verhoeven BA Verbrugh HA An antibiotic policy to prevent emergence of resistant bacilli. Lancet. 2000;355:973–978. 10768436 10.1016/s0140-6736(00)90015-1

[18] Clark RH Bloom BT Spitzer AR Empiric use of ampicillin and cefotaxime, compared with ampicillin and gentamicin, for neonates at risk for sepsis is associated with an increased risk of neonatal death. Pediatrics. 2006;117:67–74. 16396862 10.1542/peds.2005-0179

[19] Aliaga S Clark RH Laughon M Changes in the incidence of candidiasis in neonatal intensive care units. Pediatrics. 2014;133:236–242. 24446441 10.1542/peds.2013-0671 PMC3904270

[20] Fanaroff AA McDonald SA Oh W Neonatal candidiasis among extremely low birth weight infants: risk factors, mortality rates, and neurodevelopmental outcomes at 18 to 22 months. Pediatrics. 2006;117:84–92. 16396864 10.1542/peds.2004-2292

[21] Benjamin DK Jr DeLong ER Steinbach WJ Empirical therapy for neonatal candidemia in very low birth weight infants. Pediatrics. 2003;112(3 Pt 1):543–547. 12949281 10.1542/peds.112.3.543

[22] Yu Y Du L Yuan T Risk factors and clinical analysis for invasive fungal infection in neonatal intensive care unit patients. Am J Perinatol. 2013;30:589–594. 23277386 10.1055/s-0032-1329688

[23] Bahuwayrith F Detection and prevalence of extended-spectrum beta-lactamases phenotypes in Saudi Arabia. https://www.researchgate.net/publication/310844905_Detection_and_Prevalence_of_Extended-Spectrum_Beta-Lactamases_Phenotypes_in_Saudi_Arabia. Accessed February 25, 2019.

[24] Tawfik AF Alswailem AM Shibl AM Prevalence and genetic characteristics of TEM, SHV, and CTX-M in clinical Klebsiella pneumoniae isolates from Saudi Arabia. Microb Drug Resist. 2011;17:383–388. 21612509 10.1089/mdr.2011.0011

[25] Khanfar HS Bindayna KM Senok AC Extended spectrum beta-lactamases (ESBL) in Escherichia coli and Klebsiella pneumoniae: trends in the hospital and community settings. J Infect Dev Ctries. 2009;3:295–299. 19759493 10.3855/jidc.127

[26] Elkersh T Marie MA Al-Sheikh YA Prevalence of fecal carriage of extended-spectrum- and metallo-β-lactamase-producing gram-negative bacteria among neonates born in a hospital setting in central Saudi Arabia. Ann Saudi Med. 2015;35:240–247. 26409799 10.5144/0256-4947.2015.240 PMC6074465

[27] Dong Y Speer CP Late-onset neonatal sepsis: recent developments. Arch Dis Child Fetal Neonatal Ed. 2015;100:F257–F263. 25425653 10.1136/archdischild-2014-306213 PMC4413803

[28] West BA Peterside O Sensitivity pattern among bacterial isolates in neonatal septicaemia in port Harcourt. Ann Clin Microbiol Antimicrob. 2012;11:7. 22449249 10.1186/1476-0711-11-7 PMC3355022

[29] Roy I Jain A Kumar M Bacteriology of neonatal septicaemia in a tertiary care hospital of northern India. Indian J Med Microbiol. 2002;20:156–159. 17657057

